# Could Cerebrospinal Fluid Biomarkers Offer Better Predictive Value for Spinal Cord Ischaemia Than Current Neuromonitoring Techniques During Thoracoabdominal Aortic Aneurysm Repair - A Systematic Review

**DOI:** 10.21470/1678-9741-2018-0375

**Published:** 2019

**Authors:** Amer Harky, Matthew Fok, Holly Fraser, Callum Howard, Lara Rimmer, Mohamad Bashir

**Affiliations:** 1 Department of Cardiothoracic Surgery, Liverpool Heart and Chest Hospital, Liverpool, UK.; 2 Department of Vascular Surgery, Royal Liverpool Hospital, Liverpool, UK.; 3 School of Medicine, University of Liverpool, Liverpool, UK.; 4 Faculty of Biology, Medicine and Health, University of Manchester, Manchester, UK.; 5 Department of Aortovascular Surgery, Manchester Royal Infirmary, Manchester, UK.

**Keywords:** Spinal Cord Ischemia, Thoracic Aortic Aneurysm, Lactic Acid, Cytokines, Biomarkers

## Abstract

**Objective:**

Cerebrospinal fluid (CSF) drainage is a technique that has significantly reduced the incidence of spinal cord ischaemia (SCI). We present results of a systematic review to assess the literature on this topic in relation to thoracoabdominal aortic aneurysm repair (TAAR).

**Methods:**

Major medical databases were searched to identify papers related to CSF biomarkers measured during TAAAR.

**Results:**

Fifteen papers reported measurements of CSF biomarkers with 265 patients in total. CSF biomarkers measured included S-100ß, neuron-specific endolase (NSE), lactate, glial fibrillary acidic protein A (GFPa), Tau, heat shock protein 70 and 27 (HSP70, HSP27), and proinflammatory cytokines. Lactate and S-100ß were reported the most, but did not correlate with SCI, which was also the case with NSE and TAU. GFPa showed significant CSF level rises, both intra and postoperative in patients who suffered SCI and warrants further investigation, similar results were seen with HSP70, HSP27 and IL-8.

**Conclusions:**

Although there is significant interest in this topic, there still remains a significant lack of high-quality studies investigating CSF biomarkers during TAAR to detect SCI. A large and multicentre study is required to identify the significant role of each biomarker.

**Table:** 

Abbreviations, acronyms & symbols				
CSF	= Cerebrospinal fluid		MeSH	= Medical subject headings
DTAAR	= Descending thoracic aortic aneurysm repair		NSE	= Neuron-specific endolase
GFPa	= Glial fibrillary acidic protein A		OR	= *Odds ratio*
HSP	= Heat shock protein		SCI	= Spinal cord ischaemia
IONM	= Intraoperative neurophysiological monitoring		SSEP	= Somatosensory evoked potentials
LD	= Lactate dehydrogenase		TAAR	= Thoracoabdominal aortic aneurysm repair
MEPs	= Motor evoked potentials		TNF-α	= Tumor necrosis fator-alpha

## INTRODUCTION

Spinal cord ischaemia (SCI) is a major complication post open thoracoabdominal aortic aneurysm repair (TAAR). The presence of TAAR is a major risk factor for sudden death, it carries a mortality rate of 90% at 5 years if remained unoperated^[1,2]^. Surgical repair can offer a good prognosis compared to conservative management, however, such type of surgery is associated with serious morbidities perioperatively and mortality^[3]^.

One of the major perioperative complications is the development of paraplegia, which is not only physically decapitating but also can lead to serious psychological issues. Such complication is consequence of spinal cord ischaemia, which comes from limited spinal cord blood flow during surgery, for which reports as high as 30% have been described^[4]^. To protect against such complication, various adjunctive techniques have been developed to maintain appropriate spinal cord circulation and reduce the incidence of spinal cord ischaemia, among such techniques are: cerebrospinal fluid (CSF) drainage to reduce CSF pressure and increase spinal cord perfusion^[5]^, deep hypothermia which decreases the body’s metabolic activity and oxygen demand^[6]^, sequential clamping and reattachment of intercostal arteries^[7]^, and distal aortic perfusion^[8]^.

A leading technique these days to assess spinal cord function is measuring motor evoked potentials (MEPs) intraoperatively, which potentially gives surgeons a real-time assessment of spinal cord function^[9,10]^. This method is used to alert surgeons to identify impending spinal cord ischaemia and therefore have a chance to change their intraoperative strategy to prevent postoperative paraplegia. However, in current literature, there is much debate and uncertainties as to the usefulness and application of MEPs and hence some centres do not routinely use it and yet, they report low rates of paraplegia^[11]^. There does not currently exist any high-level evidence to show that the use of MEPs significantly decreases the incidence of postoperative spinal cord ischaemia.

Current evidence from the literature suggest that there is release of specific biomarkers into the CSF when there an ischaemic insult to brain or spinal cord^[12-16]^. This has been extrapolated into thoracoabdominal aneurysm repair because CSF drainage catheter is routinely applied in all thoracoabdominal aneurysm elective repairs and there are certain research outputs suggesting that there is an association of their appearance concurrently with spinal cord ischaemia. Our current research is in the development of a novel microwave sensor that can detect specific biomarkers in the CSF in real time, that we believe could offer a better real-time assessment of the spinal cord intra and postoperative thoracoabdominal aneurysm repair. In this review, we have systematically collated all published information on the measured biomarkers in the CSF during thoracoabdominal aneurysm repair and their relationship to SCI.

## METHODS

### Search Strategy

Electronic searches were performed in PubMed, MEDLINE, EMBASE, Google Scholar, SCOPUS, and Cochrane. No limits were placed on dates. Limits were placed on articles published in the English language only. Search terms used included thoracic aortic aneurysm OR thoracic aneurysm OR TAAR OR thoracic dissection OR thoracic aorta OR thoracic aortic dissection AND intrathecal OR cerebrospinal fluid OR CSF OR biomarker AND spinal cord ischaemia OR paraplegia OR paralysis OR neurological deficit. To achieve maximum sensitivity, all search terms were combined with Boolean operators and searched as both keywords and MeSH (Medical Subject Headings) terms. Following exclusion of articles based on title or abstract, full text articles selected had reference lists searched for any potential further articles to be included in this review.

## RESULTS

In total, we found 15 papers that reported measurements of CSF biomarkers following thoracoabdominal aneurysm repair. Those papers are summarized in Table 1. Biomarkers measured included S-100β, NSE, lactate, LD, TNF-α, GFAp, Tau, glucose, HSP70, HSP27 interleukin 1β, IL-6, IL-8, IL-10, IL-12, and excitatory amino acids^[17-30]^.

**Table 1:** Characteristics of studies included.

Author	Year	Country	Total number of patients	Number of male patients (%)	Average age (range)	Number with neurological deficits	CSF biomarker studied
Lases et al.^[17]^	2005	Netherlands	69	26 (55)	64 (35-89)	4 (5.8)	S-100B, NSE, LD
Casiraghi et al.^[24]^	2011	Italy	16	10 (62.5)	63 (38-75)	4 (26.7)	Lactate
Drenger et al.^[25]^	1997	Jerusalem, Baltimore and Nashville	21	N/A	65.3	3 (14.3)	Lactate
Brunnekreef et al.^[18]^	2007	Netherlands	8	3 (37.5)	68 (58-76)	0	S-100B
Kunihara et al.^[20]^	2001	Japan	15	9 (60)	60.6 (26-75)	1 (6.7%)	TNF-α, IL-1β, IL-6, IL-8, IL-10, IL-12
van Dongen et al.^[19]^	1998	Netherlands	8	7 (87.5)	63 (35-75)	1 (12.5)	S-100B
Kunihara et al.^[20]^	2001	Japan	23	12 (52.2)	64	4 (17.4)	S100-B
Anderson et al.^[21]^	2003	Sweden	11	N/A	64	2 (18.2)	S100-B, NSE, GFAp, lactate
Shiiya et al.^[22]^	2004	Japan	28	16	64 (26-79)	6 (21.4)	S100-B, Tau
Khaladj et al.^[23]^	2008	Germany	13	8	63 (27-71)	2	S100-B, lactate, glucose
Hecker et al.^[26]^	2008	Philadelphia	37	20	N/A (40-80)	14	HSP70 and HSP27
Brock et al.^[28]^	1997	Baltimore	16	6	68.5	4 (25)	Glutamate, aspartate and glycine

### S-100β

Seven studies reported levels of S-100β in the CSF as well as in patients who suffered from SCI and compared to controls (summarized in Table 2)^[17-23]^. The results are ambiguous as to whether high level of S-100β was associated with SCI or can it be used as a marker to predict SCI intraoperatively as levels of S-100β were not significantly higher in two studies for patients who suffered from SCI compared to control group^[17]^. In most studies, the rise seen in S-100β as a late change in the CSF and hence not useful as early predictive factor for SCI.

**Table 2:** Studies that have analysed S-100β in CSF as comparative analysis.

Author	Number of patients	Number with neurological deficit (%)	Mean baseline S100b concentration	Peak S100b concentration in paraplegic group (µg/l-1)
Lases et al.^[17]^	69	4 (5.8)	5.625 µg/litre	10.375
Brunnekreef et al.^[18]^	8	0	1.3 µg/litre	n/a
van Dongen et al.^[19]^	8	1(12.5)	1.23 µg/litre	10.03
Kunihara et al.^[20]^	23	4 (17.4)	1.5 µg/litre	275
Anderson et al.^[21]^	11	2 (18.2)	3.7 (1 [in log ng/l])	4.4 (in log ng/l)
Shiiya et al.^[22]^	28	6 (21.4)	1.60	42.35*
Khaladj et al.^[23]^	13	2	1.3	19.4[±14]

* Original values 72.2 [±40.8] and 12.5 [±5.5].

Lases et al.^[17]^ described a prospective clinical study of 69 patients undergoing thoracoabdominal aneurysm repair were CSF samples taken at times of induction of anaesthesia, application of aortic cross-clamping, 5 min, then 2, 4, 6, 8 and 19 hours after reperfusion. These results were concomitantly compared to serum samples and motor evoked potentials from transcranial electrical stimulation and somatosensory evoked potentials (SSEP). In this study, S-100β levels in the CSF could not be associated with silent neurological damage or adverse neurological complications postoperatively. However, CSF S-100β was higher in 75% of patients who suffered adverse neurological complications postoperatively than the 90^th^ percentile of patients without neurological complications. The median concentration of CSF S-100β remained virtually unchanged and within reference range without adverse neurological complications.

Van Dongen et al.^[19]^ in their study described the measurements of CSF S-100β in 8 patients undergoing open thoracoabdominal aneurysm repair. CSF samples in those patients were collected at time of induction, application of cross-clamping to aorta, 5 minutes following reperfusion, at skin closure, and 24 hours postoperatively. Among those 8 patients operated on, one suffered from delayed-onset postoperative paraplegia, and it was noted that CSF S-100β continued to rise from skin closure and 24 hours postoperatively, unlike the other 7 patients in whom S-100β decreased following skin closure. In the patient with postoperative paraplegia, the MEP and SSEP measurements intraoperatively did not show any significant abnormalities to indicate presence of SCI.

Kunihara et al.^[20]^ prospectively studied 23 patients undergoing TAAR or descending thoracic aortic aneurysm repair (DTAAR) who all had spinal cord drainage and CSF measurement of S-100β, the samples were taken at time of induction, 0, 6, 12, 18, 24, 48, and 72 hours postoperatively, among those 23 patients, four of them suffered from SCI (paraplegia or paralysis). In this study, S-100β levels were statistically increased 6 hours postoperatively in those with SCI compared to those without evidence of SCI (*P*<0.0010). These levels peaked at 48 hours (289 ± 454 µg/L). In this study, there was a clear correlation between S-100β CSF concentrations and SCI and the authors concluded that S-100β seems to be a sensitive reliable biomarker to predict SCI.

Anderson et al.^[21]^ investigated 11 patients undergoing elective DTAAR or TAAR and measured CSF S-100β level and furthermore measured the concentrations of its isoforms (S-100A1B and S-100BB). Two out of eleven patients suffered from postoperative neurological damage (1 stroke and 1 paraplegia). The patient who suffered from paraplegia developed this complication 3 days postoperatively. In this patient, the S-100β preoperative levels rose 20 hours after surgery and peaked (five folds higher than preoperative value) on the 3^rd^ postoperative day.

Khaladj et al.^[23]^ studied 13 patients undergoing TAAR and measured their CSF S-100β level. CSF samples were taken for analysis at time of induction, before and after application of aortic cross-clamping, following this every 10 minutes intraoperatively and then every 30 minutes, once on intensive care, samples were taken 3 times, then twice daily until removal of the CSF drain. Among the 13 patients, two suffered from spinal cord, one of them suffered severe cerebral ischaemia, as well as signs of SCI, peak CSF S-100β was 400.8 µg/L on the first postoperative day, while CSF S-100β levels continued to rise from the beginning of the operation in those with SCI, but with highest levels seen at 6 hours.

Brunnekreef et al.^[18]^ studied 8 patients who underwent thoracic endovascular stent grafting of the descending aorta. The patients were deemed at high risk of suffering postoperative paraplegia as the aneurysm involved critical intercostal arteries. CSF measurements were taken at induction, during stenting, 6 hours following completion and 20 hours after repair. In this study, no patient developed neurological deficit following graft stenting, their baseline CSF S-100β concentrations were 0.9 to 1.7 µg/L.

Shiiya et al.^[22]^ describes 28 patients undergoing DTAAR or TAAR and measurements of CSF S-100β. In this study, 3 patients developed SCI and a further patient developed both SCI and brain infarction. In the latter, CSF S-100β peaked at 965 µg/ml 48 hours postoperatively. A subsequent patient with brain infarction without SCI showed a CSF S-100β peak of 16.8 µg/ml. The other 3 patients who suffered SCI showed statisically significant higher levels of CSF S-100β from 12 to 24 hours postoperatively (median of 29.2 µg/ml and 72.2 µg/ml, respectively). Interestingly, Shiiya et al. describes 3 patients who suffered from temporary neurological deficit, their CSF S-100β rose following the completion of surgery similar to the SCI group and above those without neurological injury, however, levels of CSF S-100β decreased 24 hours postoperatively to near baseline.

The current literature investigating the association of CSF S-100β to SCI is limited. Furthermore, the results displayed are equivocal as to whether this is a potential useful biomarker. The largest of the studies (Lases et al.^17^) found that CSF S-100β did rise in 75% of those with SCI, yet remained unchanged in the other 25% of the patients. However, in the rest of studies, CSF S-100β rose in all patients who suffered from SCI, interestingly the peak level of CSF S-100β was extremely variable between studies^[17,18,20]^. For the most of the studies, increase in CSF S-100β was a late marker of SCI and was noted following completion of the operation, although a large majority of patients in these studies suffered from late-onset postoperative SCI.

### Lactate

Five studies have reported intraoperative lactate levels during thoracoabdominal aneurysm repair^17,21-25)]^, however, only three studies compared lactate CSF levels in patients with neurological deficit compared to controls (Table 3)^[23-25]^. In these patients, the lactate levels were significantly higher in patients that developed SCI postoperatively. It is also imperative to mention that when lactate was measured concomitantly with other biomarkers, it was the first biomarker to raise^[17,21,23]^, nevertheless, such raise in lactate level was non-specific as it was noted to raise at time of aortic cross-clamp application in patients without developing SCI postoperatively.

**Table 3:** Studies that have analysed lactate in CSF as comparative analysis.

Author	Number of patients	Number with neurological deficit (%)	Mean baseline lactate concentration	Peak lactate concentration in paraplegic group
Lases et al.^[17]^	69	4 (5.8)	17.75 µg/l	25.625 µg/l
Anderson et al.^[21]^	11	2 (18.2)	1.5 µg/l	3 µg/l
Khaladj et al.^[23]^	13	2	2.2 µg/l	9.3 µg/l

Lases et al.^[17]^, in the largest study of its kind, measured CSF lactate in 69 patients undergoing DTAAR or TAAR. Among the 69 patients included in the study, 4 developed adverse neurological complications. In this study, lactate did not significantly rise in all 4 patients who developed neurological complications and remained below the 90^th^ percentile of patients without adverse neurological outcomes.

Drenger et al.^[25]^ reported 21 patients who underwent TAAR and described CSF lactate measurements intra and postoperatively. Among the group, 3 patients developed paraplegia, the mean CSF lactate in such patients were significantly higher than those without evidence of SCI after removal of the aortic cross-clamp, although this difference was not noted while the cross-clamp is on.

Anderson et al.^[21]^ analysed CSF lactate level in 11 patients that underwent DTAAR or TAAR, among those 11 patients, only one patient suffered from postoperative paraplegia and the lactate CSF was high after bypass was discontinued and the level remained elevated thereafter. However, this pattern was also seen in some asymptomatic patients^[23]^, as in a study by Khaladji et al.^[23]^ who reported high CSF lactate level in 13 patients that underwent DTAAR or TAAR while only two 2 patients suffered SCI.

Casiraghi et al.^[24]^ demonstrated that in 4 of 16 patients who developed neurological injury following TAAR, CSF lactate was significantly higher at all-time points including baseline, intra and postoperatively.

### Neuron-Specific Enolase (NSE)

Neuron-specific enolase is a 78 kD gamma-homodimer and represents the dominant enolase-isoenzyme found in neuronal and neuroendocrine tissues. Its levels in other tissues, except erythrocytes, are negligible. The biological half-life of NSE in body fluids is approximately 24 hours. A comparative NSE level in CSF was measured in two studies (summarized in Table 4). Lases et al.^[17]^ compared NSE to standard MEP monitoring in their study. In their group of patients, 4 suffered postoperative paraplegia. However, the association between CSF levels of NSE and postoperative paraplegia was poorly correlated, among those patients that suffered SCI, 50% had levels of NSE greater than the 90^th^ percentile of patients with no adverse neurological outcomes. The rise in CSF levels of NSE was at late stage in the postoperative course (19 hours) and did not add value to the data obtained from MEPs. The second study was conducted by Anderson et al.^[21]^. In this study, the NSE level in patient with paraplegia was at the upper limit throughout the procedure and this did not change significantly during the entire procedure, however this was non-specific as the same increase was noted in patients who did not develop any evidence of SCI, on the contrary, it was noted that one of the patients who developed stroke postoperatively had a high level of serum NSE which peaked at first postoperative day. Ultimately, their study shows poor correlation between CSF and serum NSE value and, thus, prediction of SCI (r=0.56, *P*=0.05).

**Table 4:** Studies that have analysed NSE in CSF as comparative analysis.

Author	Number of patients	Number with neurological deficit (%)	Mean baseline NSE concentration	Peak NSE concentration in paraplegic group
Lases et al.^[17]^	69	4 (5.8)	12.625 µg/l	19 µg/l
Anderson et al.^[21]^	11	2 (18.2)	12 µg/l	17 µg/l

### Tau

Tau is the major microtubule-associated protein (MAP) of a normal mature neuron, it has two major functions, its ability to promote assembly and to maintain structure of microtubules of the neurons, and therefore derangements in tau can cause serious neurological issues.

In a study by Shiiya et al.^[22]^, they reported the levels of Tau protein in CSF for 28 patients that underwent DTAAR or TAAR (summarized in Table 5). In their study, incidence of postoperative spinal cord ischaemia was recorded in 4 of the 28 patients and TAU was significantly associated with brain infarction, but it failed to rise at all or detect spinal cord injury when compared to controls in this group of patients. Therefore, use of such biomarker remains unpredictive for SCI during open aortic repair surgeries.

**Table 5:** Study that have analysed Tau level in CSF as comparative analysis.

Author	Number of patients	Number with neurological deficit (%)	Mean baseline TAU concentration	Peak Tau concentration in paraplegic group
Shiiya et al.^[22]^	28	6 (21.4)	134.25 pg/ml	989 pg/ml (massive confidence intervals)

### Heat Shock Proteins (HSP)

Heat shock proteins are a group of proteins that are produced in response to stressful conditions; initially they were described in response to heat shock, however later they were noted in other stressful tissue situations. A study by Hecker et al.^[26]^ reported measurements of HSPs (HSP70 and HSP27) in the CSF of 37 patients that underwent TAAR. CSF samples were taken at baseline, at aortic cross-clamping, 1, 2, 12, 24 hours after cross-clamping, at the completion of surgery, and if there were any signs of paraplegia. Fifteen patients were reported as having postoperative paraplegia. In these patients the cumulative averages and mean values of both HSP70 and HSP27 were larger than patients who did not develop paraplegia. These values became statistically significant at 12 and 24 hours postoperatively. Furthermore, they showed that patients with non-linear HSP measurements overtime or had larger positive average changes from pre- to post-cross-clamp time were more likely to experience paraplegia.

### Glial Fibrillary Acidic Protein (GFAp)

Glial fibrillary acidic protein is an intermediate filament protein that is expressed by many cell types of the central nervous system. First described in 1971^[29]^, GFAp is thought to help in maintaining astrocyte mechanical strength, however, it was first named, isolated and characterized by Eng et al. in 1969^[30]^.

Anderson et al.^[21]^ reported the only study in which GFAp was measured in 11 patients that underwent DTAAR or TAAR (summarized in Table 6). In this study, one patient suffered delayed SCI and showed a 270-fold increase from baseline in CSF GFAp. In this patient, paraplegia developed 30 hours after surgery; GFAp levels were elevated before and after cardiopulmonary bypass, increasing 3-fold between 1 and 6 hours after surgery to 270-fold at 20 hours. On the contrary, one patient showed raised postoperative GFAp levels despite no adverse neurological events being noted.

**Table 6:** Study that have analysed GFAp level in CSF as comparative analysis.

Author	Number of patients	Number with neurological deficit (%)	Mean baseline GFAp concentration	Peak GFAp concentration in paraplegic group
Anderson et al.^[21]^	11	2 (18.2)	2.8	5

### Glucose

CSF glucose was measure during DTAAR or TAAR by Khaladj et al.^[23]^. Two patients developed SCI and glucose concentration was slightly increased in the SCI. However, this was not statistically significant and did not correlate well to the occurrence of SCI.

### Inflammatory Cytokines

Kunihara et al.^[27]^ reported the CSF levels of proinflammatory cytokines of 15 patients who underwent TAAR. Such proinflammatory cytokines included TNF-α, IL-1β, IL-6, IL-8, IL-10 and IL-12, which were measured at 0, 6, 12, 18, 24, 48 and 72 hours postoperatively. Among those 15 patients there was one patient that suffered paraplegia, while the remaining 14 non-paraplegic cases CSF IL-8 peaked postoperatively and continued to decline up to 72 hours postoperatively. In contrast, the patient with paraplegia IL-8 CSF rose postoperatively in consistent with the other 14 patients, however, IL-8 levels remained significantly high with no indication of decrease 72 hours postoperatively. CSF IL-6 and TNF-α did not show any significant changes throughout the study period. Interleukin-1β and IL-12 were undetectable in all 15 patients and therefore could not be used as a guide for predicting SCI.

### Excitatory Amino Acids

The study by Brock et al.^[28]^ is the only one of its kind to report levels of amino acids glutamate, aspartate and glycine in patients undergoing DTAAR or TAAR. They studied a total of 16 patients, of whom 4 displayed lower motor deficits postoperatively. They took measurements at baseline, at time of application of aortic cross-clamp, early reperfusion (within 6 hours), mid reperfusion (from 7 to 15 hours) and late reperfusion (over 15 hours). Measurements from the SCI group showed significant differences in glutamate but did not show differences in glycine level at all measurement’s times, except baseline. Aspartate showed significant increases in the SCI group during late perfusion measurements only. Glutamate levels increased significantly across all measurements; nearly threefold at time of aortic cross-clamp application (375%), remained high at early reperfusion, but lowering to around 250%, before rising at late reperfusion to approximately 400% of baseline.

## DISCUSSION

Surgical intervention remains the recommended treatment in patients with thoracoabdominal aortic aneurysms that cannot benefit from endovascular management, this option is particularly important to prevent dissection or rupture and, ultimately, death. Paraplegia remains a devastating complication of TAAR, particularly when considering that many of such patients are being operated on at elective basis. Several methods developed in light of the risk of paraplegia, such as adjuncts to surgery like deep hypothermic circulatory arrest, circulatory support, CSF drainage, and the use of different forms of intraoperative neurophysiological monitoring (IONM) in the form of MEPs and SSEP^[31]^. Those monitoring tools have a key role as an adjunct-monitoring method while repairing the diseased aorta to predict the risk of neurological injuries and provide rescue when it occurs and hence the outcomes could be better when using such methods^[31]^. In a large study by Estrera et al., of 105 complex aortic repairs CSF drainage, MEPs and SSEPs played a key role in early detection of SCIs, such injuries were noted through alterations in the signals conduction and therefore immediate actions were taken to revert this^[32]^. Similar outcomes were also reported by Dong et al. of the fact the both MEPs and SSEPs can predict spinal ischaemia and their intraoperative use can prevent the development of paraplegia^[33]^. Finally, in a systematic review and meta-analysis by Fok et al., they have reported the beneficial use of IONM including both MEPs and SSEPs, however there is lack of general consensus about the superiority or full recommendation of either method^[34]^.

CSF biomarkers represent an area that has previously undergone much research within the neurosurgical field, with particular respects to traumatic brain injury where there are several ongoing clinical trials^[35-37]^. This research has begun to trickle into the aortic world through small observational studies. Specific attention has focused on the biomarkers lactate, S-100β, NSE and GFAp, which have all shown to be good indicators correlating to injury magnitude in traumatic brain injury, as well as outcomes and survival. The most promising potential biomarkers are S-100β, GFAp and HSP, according to our analysis and published literature.

S-100β is part of the S100 protein family and the most widely studied biomarker and is highly conserved in the mammalian brain, with the majority found in large concentrations within the cytoplasm of astrocytes^[38]^. S-100β is a neurotropic dimeric acidic Ca^2+^ -binding protein with a molecular weight of 21 kDa, existing in 2 β subunits (S-100αβ heterodimer and S-100ββ homodimer). One study in this review differentiated between the two subunits but found no relevance between the two, the majority of commercially available assays do not differentiate between S-100αβ heterodimer and S-100ββ homodimer^[21,39]^.

The results for S-100β as a potential biomarker in TAAR to detect SCI have been ambiguous. The largest of the studies involving 69 patients did not find significant changes in CSF S-100β in those who suffered neurological damage^[15]^. Despite this, other smaller studies have found statistically significant associations of S-100β concentrations in CSF for patients with neurological damage^[18,20,21]^. One study described concentrations of S-100β and patients with temporary neurological damage and S-100β would rise in a fashion similar to those who suffered permanent neurological damage, but rather than plateau or continue to rise, would decrease back toward baseline^[21]^. This finding suggests that changes in S-100β concentration may reflect temporary or permanent neurological damage. However, the rise of S-100β was always a late sign of neurological damage, whether this is a reflection of sample timing could be argued.

Although only investigated in one study^[21]^, the data provided are suggestive evidence that GFAp may serve as a biomarker for SCI in TAAR, or at least warrants further investigation. GFAp is the principal 8-9 nm intermediate filament found in mature fibrillary astrocytes. Astrogliosis is the morphological and functional changes seen in astrocytes and occurs in response to a range of neurological diseases or CNS injury, including ischaemia. This process has also been shown to cause the increased expression of GFAp. Anderson et al.^[21]^ noted, combined with the relative insolubilty of GFAp, increased concentrations could reflect structural CNS damage and release into CSF would imply disintegration of cells containing GFAp.

The highly conserved large family of heat shock proteins often coined as molecular chaperones, found in all cells of all organisms, so called because of their initial discovery when their dramatic up-regulation was detected following subjection of cells to heat shock^[19]^. They play crucial roles in folding/unfolding of proteins, assembly of multiprotein complexes, transport/sorting of proteins into correct subcellular compartments, cell-cycle control and signalling, and cell protection against stress/apoptosis. Furthermore, following their discovery they are released in a variety of other stresses, including ischaemia. Hecker et al. choose to study HSP27 and HSP70 families for their association with cellular protection and recovery after a near-lethal stress^[24]^.

One of the main limiting factors in looking at the association between CSF biomarkers and SCI is that detection of these markers requires extensive and immediate laboratory testing. Consequently, this requires time, which is not ideal for intraoperative use, specific equipment, which is expensive and furthermore needs technical expertise. The ideal biomarker is one that is both sensitive and specific, that can be rapidly, inexpensively and easily detected. Furthermore, there must be good evidence to show that timely intervention in the presence of this biomarker actually changes surgical outcomes. Although the quest for a biomarker continues, it will require real-time detection to make intraoperative decisions to prevent SCI. These requisites make the search for the biomarker to detect SCI in TAAR very challenging.

## CONCLUSION

CSF may potentially hold clinically relevant information regarding SCI, however, this is an under-researched area in the thoracic aortic surgery world. So far, to the authors’ knowledge, the most promising candidates are heat shock proteins, GFAp and S-100β. Detection of these biomarkers requires both time and financial support as they have very dynamic changes in their concentrations and are hard to measure experimentally. Our future research will be in the further development of a real-time sensor that can detect small changes in the concentration of these proteins.

**Table:** 

Author's roles & responsibilities	
AH	Substantial contributions to the conception or design of the work; or the acquisition, analysis, or interpretation of data for the work; final approval of the version to be published
MF	Substantial contributions to the conception or design of the work; or the acquisition, analysis, or interpretation of data for the work; final approval of the version to be published
HF	Substantial contributions to the conception or design of the work; or the acquisition, analysis, or interpretation of data for the work; final approval of the version to be published
CH	Substantial contributions to the conception or design of the work; or the acquisition, analysis, or interpretation of data for the work; final approval of the version to be published
LR	Substantial contributions to the conception or design of the work; or the acquisition, analysis, or interpretation of data for the work; final approval of the version to be published
MB	Substantial contributions to the conception or design of the work; or the acquisition, analysis, or interpretation of data for the work; final approval of the version to be published
